# Ferulic Acid Treats Gastric Ulcer via Suppressing Oxidative Stress and Inflammation

**DOI:** 10.3390/life13020388

**Published:** 2023-01-31

**Authors:** Aleyna Ermis, Gozde Aritici Colak, Merve Acikel-Elmas, Serap Arbak, Meltem Kolgazi

**Affiliations:** 1 Department of Nutrition and Dietetics, Faculty of Health Science, Acibadem Mehmet Ali Aydinlar University, Icerenkoy Mah., Kayisdagi Cad. No: 32, Atasehir, Istanbul 34752, Turkey; 2 Department of Histology and Embryology, School of Medicine, Acibadem Mehmet Ali Aydinlar University, Icerenkoy Mah., Kayisdagi Cad. No: 32, Atasehir, Istanbul 34752, Turkey; 3 Department of Physiology, School of Medicine, Acibadem Mehmet Ali Aydinlar University, Icerenkoy Mah., Kayisdagi Cad. No: 32, Atasehir, Istanbul 34752, Turkey

**Keywords:** gastric ulcer, indomethacin, antioxidant, anti-inflammatory, ferulic acid

## Abstract

(1) Background: The aim of the present study was to evaluate the gastroprotective potential of ferulic acid (FA) on indomethacin-induced gastric ulcers in rats with macroscopic and microscopic examinations along with biochemical assays. (2) Methods: After 24 h starvation, the ulcer was induced in male Sprague-Dawley rats by subcutaneous indomethacin (25 mg/kg) injection. Fifteen minutes after ulcer induction, rats were treated with either tween 80 or FA. FA was given by oral gavage at 100 mg/kg, 250 mg/kg, and 500 mg/kg. In the fourth hour, rats were euthanized and collected gastric samples were evaluated macroscopically and microscopically. Antioxidant parameters including malondialdehyde (MDA), glutathione (GSH), superoxide dismutase (SOD), and inflammatory parameters comprising of myeloperoxidase (MPO), Tumor Necrosis Factor (TNF)-α, Interleukin (IL)-1β, IL-6 and Nuclear Factor Kappa-B (NF-κB) p65 levels were also determined. (3) Results: Indomethacin injection significantly increased the macroscopic and microscopic scores. In addition, it increased the gastric MDA, MPO, TNF-α, IL-1β, IL-6, and NF-κB p65 levels but reduced SOD and GSH content. Treatment with FA significantly improved the gastric injury macroscopically and microscopically. Moreover, FA displayed a marked decrease in the gastric levels of MDA, MPO, TNF-α, IL-1β, IL-6, and NF-κB p65 and a significant increase in SOD and GSH compared to the INDO group. Ultimately, 250 mg/kg FA was determined as the most effective dose. (4) Conclusion: Our results revealed that FA has a gastroprotective effect against indomethacin-induced gastric ulcers in rats due to its antioxidant and anti-inflammatory properties. As a result, FA may be a potential treatment choice for gastric ulcers.

## 1. Introduction

Gastric ulcer is a digestive system disease characterized by a lesion of the gastric tissue’s mucosal integrity from the muscularis mucosa to the submucosa or deeper, affecting 5–10% of the world’s population [1]. Under normal circumstances, mucus and bicarbonate (HCO_3_^−^), which function as a protective layer, are released by mucosal cells to neutralize hydrochloric acid (HCl) [2]. The etiology of gastric ulcers is unknown, but the main mechanism underlying its development is an imbalance between protective (tight junctions between epithelial cells, microcirculation, HCO_3_^−^ production, mucus secretion, adequate level of prostaglandins and nitric oxide) and aggressive factors (increased gastric acid secretion, pepsin activity, bile salt secretion, ethanol) [3]. Gastric ulcer is a polyetiological and polypathogenetic disease because of the various mechanisms underlying ulceration [4].

NSAIDs contain analgesic, antipyretic, and anti-inflammatory properties and are among the most extensively used medications for the treatment of inflammatory illnesses such as fever, acute or chronic pain, and rheumatic diseases across the world [5]. However, one of the distinct adverse effects of NSAIDs is mucosal damage in the stomach. The inhibition of the COX enzyme, which produces thromboxane and prostaglandins, is the principal mechanism by which NSAIDs induce ulceration and gastrointestinal problems. NSAIDs inhibit the COX pathway, lowering prostaglandin synthesis, which protects the stomach mucosa by boosting mucus and HCO_3_^−^ release, as well as enhancing mucosal blood flow [6]. Because it produces substantial damage to the stomach tissue, indomethacin, which inhibits both isoforms of the COX enzyme, is one of the first-choice drugs for experimental ulcer models. Inhibition of protective factors such as PGE2, gastric mucosal blood flow, HCO_3_^−^, and mucus, imbalance of oxidative and anti-oxidative states, lipid peroxidation, increased production of inflammatory biomarkers, and inflammatory cell infiltration with neutrophil activation are also crucial mechanisms involved in the pathogenesis of indomethacin-induced gastric ulcers [7].

Recently, proton pump inhibitors, histamine type-2 receptor antagonists, antacids, and antibacterial treatments for H. pylori infections have been utilized in clinical practice to repair the protective layer and reduce the quantity of stomach acid. Although each medicine has its own pharmacology, the common feature of these drugs is that they inhibit gastric acid secretion or stimulate HCO_3_^−^ and mucus secretion by blocking H2 receptors/gastric hydrogen/potassium ATPase (H^+^-K^+^-ATPase). Arrhythmia, gynecomastia, and hematological abnormalities are all possible adverse effects of long-term usage of these drugs [8]. Because the pathogenesis of a gastric ulcer is complex, it has become a research target to identify safe, effective, and non-toxic agents that can increase the gastric mucosa’s endogenous defense capability, as well as lower inflammation and stomach acid secretion, which is critical for gastric ulcer treatment [9].

Ferulic acid (FA) is a hydroxycinnamic acid that is mainly found in rice, apples, barley, oranges, coffee, wheat, and peanuts [10]. Gastric absorption of FA is rapid and passes into the small intestine unaltered by stomach acid. The half-life of FA is approximately 30 min in rats, depending on the dose and route of administration, and therefore it has low toxicity [11]. In addition to having low toxicity, it shows anti-inflammatory, antioxidant, antidiabetic, antithrombotic, antimicrobial, antiallergic, antiviral, neuroprotective, and anticancer (lung, breast, colon, and skin cancer) activities [12]. In various pathophysiological conditions, FA acts as an anti-inflammatory agent by decreasing proinflammatory mediators such as TNF-α, IL-6, and IL-1β, increasing anti-inflammatory cytokines and modulating cell signaling pathways [13]. FA is a critical modulator of the proinflammatory cytokine signaling pathway and reduces the expression of NF-κB, which is involved in oxidative stress and inflammatory responses [14].

A previous study has reported the gastroprotective effects of various phenolic acids, including ferulic, caffeic, p-coumaric, and cinnamic acids, against ethanol-, indomethacin-, or stress-induced acute gastric ulcers due to their resulting in a reduction in the lesion index, the total area of the lesion, the percentage of the lesion, volume of gastric juice, and total acidity [15]. However, relevant hydroxy cinnamic acids have been evaluated, but the role of FA’s anti-ulcer potential on the macroscopic score as well as on oxidative stress and inflammation remained unknown. Therefore, the purpose of this study was to evaluate the anti-ulcerogenic properties of FA against indomethacin-induced stomach ulcers in depth.

## 2. Materials and Methods

### 2.1. Animals

Adult male Sprague-Dawley rats (*n* = 40), weighing 200 to 250 g, were obtained from the Laboratory Animal Application and Research Center, Acibadem Mehmet Ali Aydinlar University, Istanbul, Turkey. The animals were housed under standard conditions of temperature (22 ± 2 °C), humidity (65–70%) and 12 h light (12 h/12 h light/dark cycles) and were fed with a standard pellet diet ad libitum (Optima, Sincan, Turkey) and allowed free access to water. Animals were grouped and housed randomly into ten wire-mesh cages (four rats each), where they acclimatized to the housing circumstances for one week prior to the commencement of the experiment.

### 2.2. Ethical Consideration

All the experimental procedures were carried out following the guidelines for the care and handling of laboratory animals as recommended by the National Institutes of Health (NIH 85–23). Experimental protocols and procedures used in this study were approved by the Local Ethics Committee for Animal Experiments of Acibadem Mehmet Ali Aydinlar University, Istanbul (ACU-HADYEK 2020/26).

### 2.3. Drugs and Other Chemicals

Indomethacin (Cayman Chemical, Ann Arbor, MI, USA) was dissolved in 5% NaHCO_3_ and was administered to the animals subcutaneously. Ferulic acid (Sigma Aldrich, Taufkirchen, Germany) was dissolved in 10% tween 80 (Merck, Darmstadt, Germany) and then was administered to the rats with oral gavage.

### 2.4. Indomethacin-Induced Gastric Ulcer

Gastric ulceration was induced in rats according to the procedure [16]. Briefly, the rats were kept fasting for 24 h before the experiment with free access to water ad libitum and were placed in cages with raised wide mesh floors to prevent coprophagy. Then, indomethacin was administered as a single dose (25 mg/kg dissolved in 5% NaHCO_3_, subcutaneously) to induce gastric ulcers.

### 2.5. Experimental Design

Rats were randomly divided into five groups, each consisting of eight rats: the control group, the INDO group and, the FA groups (FA100, FA250, and FA500 groups). All groups received subcutaneous indomethacin 25 mg/kg dissolved in 5% NaHCO_3_, except the control group. The control group received an identical volume of saline. Fifteen minutes later, the FA groups were treated with FA 100 mg/kg, 250 mg/kg, and 500 mg/kg (suspended in 1 mL of 10% tween 80), respectively, by oral gavage. Doses of ferulic acid were determined considering previous studies, including our group’s preliminary research [15,17,18]. On the other hand, the control and INDO groups were given vehicle (10% tween 80) by oral gavage. Four hours after the administration of indomethacin, animals were euthanized by exsanguination under isoflurane anesthesia. The stomachs were dissected out and incised along the greater curvature. They were then rinsed with cold 0.9% physiological saline and collected for macroscopic examination, microscopic scoring, histological evaluation, and biochemical analyses. Stomach samples were examined macroscopically and then stomachs were made into two parts. A piece of gastric tissue was taken from each stomach and fixed with 10% formaldehyde for microscopic scoring, histological evaluation, and transmission electron microscopy, and the rest of the gastric tissue was stored at 80 °C for biochemical analysis of oxidative stress and inflammatory markers [myeloperoxidase (MPO), malondialdehyde (MDA), glutathione (GSH), superoxide dismutase (SOD), catalase (CAT), tumor necrosis factor (TNF)-α, interleukin (IL)-1β, interleukin 6 (IL-6), and nuclear factor kappa-B-p65 (NF-κB-p65) level].

### 2.6. Macroscopic Evaluation

The severity of macroscopic lesions developed was assessed as previously reported [19] using the following semiquantitative scale. This scale was applied for macroscopic scoring by two observers unaware of the experimental protocol and determined by measuring the length of each lesion along its greatest diameter, scoring from 0 to 3 as follows: 0: normal mucosa; 1: 1–4 small petechiae; 2: 5 or more petechiae or hemorrhagic streaks up to 4 mm long; and 3: erosion of more than 5 mm or confluent hemorrhages.

### 2.7. Estimation of Biochemical Parameters in Gastric Tissue

The stomach tissue samples from all the groups were collected, and parts of the stomach samples were cut into small pieces and then homogenized to obtain the supernatant. The gastric homogenate was used for the assessment of the following biochemical parameters:

#### 2.7.1. Measurement of Gastric Oxidative Stress Markers Levels

To assess gastric oxidative stress, MDA, GSH, and CAT levels in the gastric tissue were measured. Malondialdehyde (MDA) content in gastric tissues was tested to obtain quantified byproducts of membrane lipid peroxidation through assessing the formation of thiobarbituric acid-reactive substances as described before. When MDA is heated with TBA under acid circumstances, it forms a pink-colored substance. Then, a maximum absorbance of 532 nm was detected by a spectrophotometer (Shimadzu, UV-2600/UV-VIS/Spectrophotometer) and the results were expressed as nanomoles of MDA per gram of gastric tissue (nmol/g tissue).

Gastric enzymatic antioxidant enzymes were determined. The SOD test uses xanthine and xanthine oxidase to produce superoxide radicals, which react with 2-(4-iodophenyl)-3-(4-nitrophenol)-5-phenyltetrazolium chloride to produce a red formazan dye. The degree of inhibition of this reaction is then used to determine the SOD activity. The concentrations of SOD in the stomach tissue homogenate were measured using a commercial kit (Relassay, Turkey) and a colorimetric autoanalyzer (Mindray, BS-400). The results are reported as the quantity of SOD in U/mL.

Non-enzymatic antioxidant activity was also determined. GSH was measured using a spectrophotometric approach based on a modified Ellman procedure. Shortly, the supernatant was collected after centrifugation and added to a combination of 2 mL of 0.3 mol/l Na_2_HPO_4_–2H_2_O solution and 0.2 mL of dithiobis-nitrobenzoate solution (0.4 mg/mL in 1% sodium citrate). A spectrophotometer (Shimadzu, UV-2600/UV-VIS/Spectrophotometer) was used to determine the absorbance of the combination at 412 nm. GSH levels were expressed in micromoles per gram of tissue (μmol/g).

#### 2.7.2. Measurement of MPO Activity and Gastric Inflammatory Mediators’ Levels

To assess gastric inflammation, MPO activity and TNF-α, IL-1β, IL-6, NF-κB p65 levels in the gastric tissue were measured. The enzymatic activity of MPO was measured in gastric ulcer homogenized tissue. The H_2_O_2_-dependent oxidation of o-dianisidine 2HCl was used to measure MPO activity, which is a marker of neutrophil accumulation. The quantity of MPO present that causes changes in absorbance of 1.0 unit/min at 460 nm and 37 °C, expressed in units per g of tissue, was determined as one unit of enzyme activity. MPO activity was quantified spectrophotometrically according to the reaction. The activity of MPO was measured in units per gram of tissue (U/g). The concentrations of the proinflammatory cytokines TNF-α, IL-1β, IL-6 and NF-κB p65 in the homogenate of gastric tissue were performed using a specific rat enzyme-linked immunosorbent assay (ELISA) kit (Elabscience, Houston, TX, USA) according to the manufacturer’s instructions. The optical density (OD) was 450 nm and it was measured spectrophotometrically (ELx800 Microplate Reader, Biotek, Winooski, VT, USA). The results of TNF-α, IL-1β, IL-6, and NF-κB were expressed in picograms per millimeter (pg/mL).

### 2.8. Histological Evaluation

Tissue samples were fixed in 10% formalin and processed for routine paraffin embedding for histological observations. Tissue sections (approximately 5 μm thick) were prepared using a rotary microtome (Rotary, Thermo Scientific, Waltham, MA, USA) and stained with hematoxylin and eosin to assess histological changes by light microscopy (Zeiss Axio Scope.A1 AX10), and photographs were taken (Zeiss AxioCam MRc 5). Specimens were graded by light microscopy according to a previously reported grading method [20] that comprises the assessment of epithelial desquamation, mucosal hemorrhage, glandular damage, and inflammatory cell infiltration on a scale of 0 to 3 (0: none, 1: mild, 2: moderate, and 3: severe) for each criterion.

### 2.9. Scanning Electron Microscopy (SEM)

Tissue samples were also evaluated by scanning electron microscopy. They were immersed in 2.5% glutaraldehyde (pH 7.2) and processed for scanning electron microscopy and examined under a scanning electron microscope (Thermo Fischer, Quattro S). Basal lamina and surface epithelium integrity were examined under a scanning electron microscope (Thermo Fischer, Quattro S).

### 2.10. Statistical Analysis

Data are expressed as mean ± standard error (SE). Statistical comparisons were carried out using one-way analysis of variance (ANOVA) followed by Tukey–Kramer multiple comparison tests or Student’s *t*-test. A *p*-value of <0.05 was taken to indicate statistical significance. The data were analyzed using GraphPad Prism software, version 8 (GraphPad Software, San Diego, CA, USA). 

## 3. Results

### 3.1. Macroscopic and Microscopic Scores

**Figure 1:**
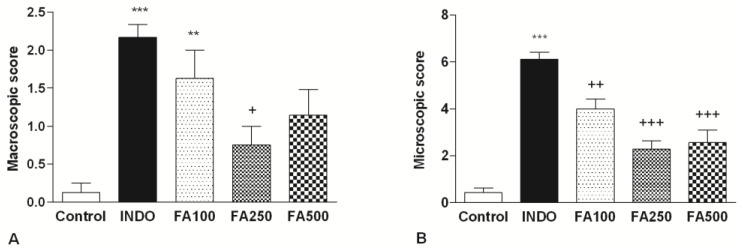
Macroscopic (**A**) and microscopic (**B**) damage scores of gastric tissue samples. INDO: Indomethacin; FA100: Indomethacin + Ferulic Acid 100 mg/kg; FA250: Indomethacin + Ferulic Acid 250 mg/kg; FA500: Indomethacin + Ferulic Acid 500 mg/kg. ** *p* < 0.01 and *** *p* < 0.001 vs. the control group; + *p* < 0.05, ++ *p* < 0.01 and +++ *p* < 0.001 vs. the INDO group.

As shown in Figure 1A, administration of indomethacin at a single dosage of 25 mg/kg resulted in the production of gastric injury and caused a significant increase (2.167 ± 0.167, *p* < 0.001) in macroscopic score as compared to the control group (0.125). There were remarkable confluent hemorrhages, red-black bleeding areas, and petechial lesions in the stomachs of the indomethacin group while treated only with the vehicle. The rats in the control group did not show gastric mucosal lesions and had a normal anatomical appearance. The anti-ulcer effect of both doses of FA on indomethacin-induced gastric damage was macroscopically determined in rats. Compared with the indomethacin group, treatment with all doses of FA (100 mg/kg, 250 mg/kg, 500 mg/kg) revealed a decrease in hyperemia, hemorrhage, and ulceration. In macroscopic scoring, it was determined that the scores had a significantly higher in the FA100 group (1.63 ± 0.38, *p* < 0.01) compared to the control group (0.125). Notably, 250 mg/kg FA (1.75 ± 0.25; *p* < 0.05) significantly decreased the macroscopic scores (1.75 ± 0.25; *p* < 0.05) in comparison to the INDO group. These results showed that 250 mg/kg FA had a significant treating effect against the gastric injury caused by indomethacin.

The gastroprotective effects of FA on stomach structural changes caused by indomethacin administration were also assessed using a light microscope to evaluate the histological examination of gastric tissues. In histological evaluation, the normal structure of the stomach was observed in the control group (Figure 1B). On the other hand, in the INDO group, indomethacin administration caused severe gastric tissue damage, and histological changes including the marked presence of surface epithelial damage, mucosal hemorrhage, and inflammatory cell infiltration were determined. According to the microscopic score evaluation of gastric lesions, it was determined that the INDO group (6.125 ± 0.295) had a significantly higher score compared to the control group (0.429 ± 0.202 *p* < 0.001). In contrast, both doses of FA showed a decrease in the histological damage caused by indomethacin and repaired gastric mucosa integrity, decreasing the mucosal hemorrhage and infiltration of inflammatory cells. It was determined that the microscopic score significantly decreased in the FA100, FA250, and FA500 groups (4 ± 0.423; *p* < 0.01, 2.286 ± 0.395; *p* < 0.001, 2.571 ± 0.528; *p* < 0.001, respectively) compared to the INDO group. In the control group, the normal structure of the gastric mucosa was observed; nevertheless, oral administration of FA also showed lower histological changes.

### 3.2. Effect of FA on MPO and Gastric Oxidative Stress Markers Levels

**Figure 2:**
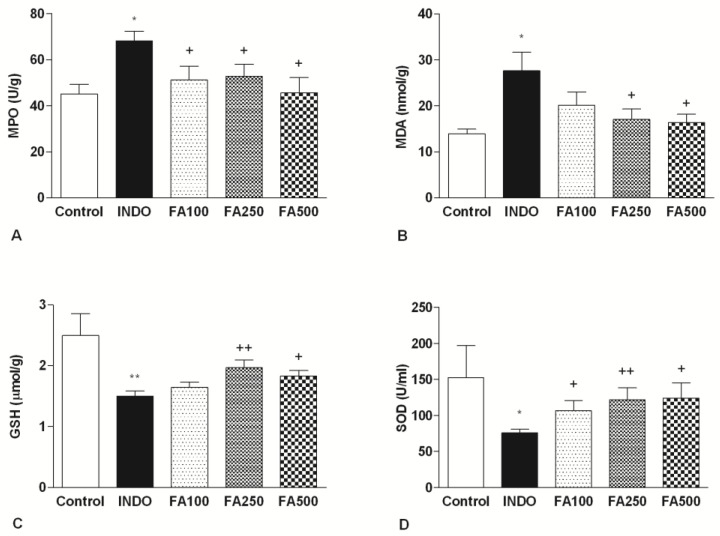
MPO (**A**), MDA (**B**), GSH (**C**) and, SOD (**D**) levels of gastric tissue samples. MDA: Malondialdehyde, MPO: Myeloperoxidase, GSH: Glutathione, SOD: Superoxide dismutase. INDO: Indomethacin; FA100: Indomethacin + Ferulic Acid 100 mg/kg; FA250: Indomethacin + Ferulic Acid 250 mg/kg; FA500: Indomethacin + Ferulic Acid 500 mg/kg. * *p* < 0.05 and ** *p* < 0.01 vs. control group; + *p* < 0.05 and ++ *p* < 0.01 vs. INDO group.).

MPO enzyme activity in gastric tissue was measured as a marker of neutrophil infiltration. The activity of MPO after administration of indomethacin led to an increase in the INDO group (68.23 ± 4.25 U/g) compared to the control group (45.22 ± 4.11 U/g; *p* < 0.05). Additionally, for all doses of FA (100 mg/kg, 250 mg/kg, 500 mg/kg), it was observed that there was a significant reduction in the levels of gastric MPO levels (respectively, 51.20 ± 5.96 U/g; *p* < 0.05, 52.91 ± 5.11 U/g; *p* < 0.05, 45, 76 ± 6.51 U/g; *p* < 0.05) (Figure 2A).

Figure 2B shows the lipid peroxidation marker, malondialdehyde (MDA), and antioxidant activity in gastric tissue samples. The gastric MDA level in the indomethacin group (27.66 ± 4.023 nmol/g), was characterized by a significant increase compared to the control group (13.91 ± 1.033 nmol/g; *p* < 0,05), as a marker of lipid peroxidation and tissue damage. While the reduction with the 100 mg/kg dose did not reach statistical significance (20.18 ± 2.856 nmol/g), the MDA levels significantly decreased with both the 250 mg/kg and 500 mg/kg FA treatments (17.1 ± 2.257 nmol/g; *p* < 0.05, 16.42 ± 1.81 nmol/g; *p* < 0.05, respectively).

Indomethacin administration caused the depletion of the endogenous antioxidant GSH content and GSH levels were lower in the INDO group (1.501 ± 0.081 μmol/g) compared to the control group (2.495 ± 0.357 μmol/g; *p* < 0.001). Treatment with 100 mg/kg FA had no significant differences against the gastric ulcer (1.643 ± 0.088 μmol/g), and treatment with 250 mg/kg and 500 mg/kg FA significantly inhibited the reduction of GSH induced by indomethacin (1.967 ± 0.128 μmol/g; *p* < 0.001, 1.829 ± 0.088 μmol/g; *p* < 0.05, respectively) (Figure 2C).

The scavenging SOD activity of superoxide radicals was lower in the INDO group (75.70 ± 5.18 U/mL) compared to the control group (152.3 ± 44.63 U/mL; *p* < 0.05). A marked increase in SOD enzymatic activity was observed in the rats treated with all doses of FA compared to the INDO group (106.7 ± 14.17 U/mL; *p* < 0.05, 122 ± 16.20 U/mL; *p* < 0.01, 124 ± 21.03 U/mL; *p* < 0.05) (Figure 2D). The most improvement in GSH content and SOD activity occurred by 250 mg/kg FA.

### 3.3. Effect of FA on Gastric Inflammatory Mediators’ Levels

**Figure 3:**
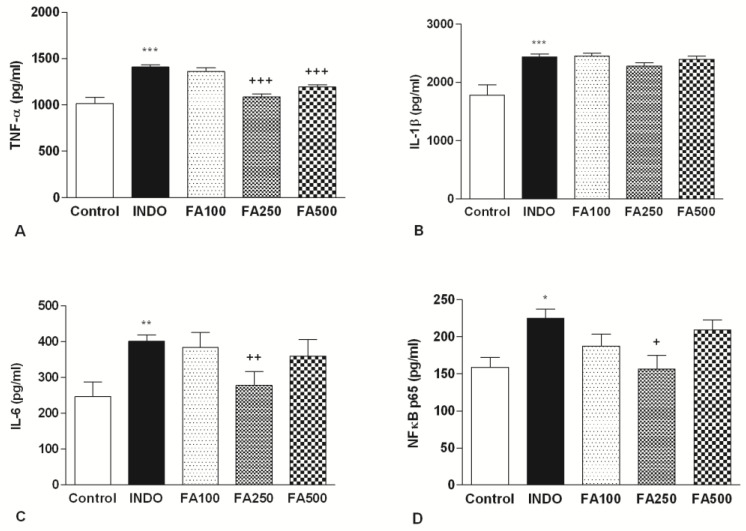
TNF-α (**A**), IL-1β (**B**), IL-6 (**C**) and, NF-κB (**D**) p65 levels of gastric tissue samples. TNF-α: Tumor necrosis factor-α, IL-1β: Interleukin-1β, IL-6: Interleukin 6, NF-κB-p65: Nuclear Factor Kappa-B-p65. INDO: Indomethacin; FA100: Indomethacin + Ferulic Acid 100 mg/kg; FA250: Indomethacin + Ferulic Acid 250 mg/kg; FA500: Indomethacin + Ferulic Acid 500 mg/kg. * *p* < 0.05, ** *p* < 0.01 and *** *p* < 0.001 vs. control group; + *p* < 0.05, ++ *p* < 0.01 and +++ *p* < 0.001 vs. INDO group.

Indomethacin administration caused a dramatic increase levels of TNF-α, a key proinflammatory cytokine involved in the formation of the gastric ulcer in the INDO group (1.409 ± 19.66 pg/mL), compared to the control group (1.016 ± 65.54 pg/mL; *p* < 0.001). TNF-α levels (1.357 ± 41.93 pg/mL) in the FA100 group were not different from the INDO group. On the other hand, doses of 250 mg/kg and 500 mg/kg FA (respectively, 1.086 ± 32.18 pg/mL; *p* < 0.001, 1.194 ± 23.49 pg/mL; *p* < 0.001) significantly decreased the level of TNF-α (Figure 3A).

Similarly, significant increase in the level of gastric IL-6 was indicated in the INDO group (400.9 ± 17.71 pg/mL) when compared to the control group (246.4 ± 40.93 pg/mL; *p* < 0.01). IL-6 levels of FA100 (384 ± 40.8) and FA500 (359.2 ± 46.55) groups were similar with the INDO group. There was a notable reduction only in the group treated with 250 mg/kg FA when compared with the INDO group (278.3 ± 38.21 pg/mL; *p* < 0.01) (Figure 3B).

Following ulcer induction, the gastric levels of IL-1β in the INDO group (2.439 ± 46.66 pg/mL) were found to be higher than the control group (1.780 ± 176.2 pg/mL); *p* < 0.001). None of the treatment doses reduce IL-1β levels (2.451 ± 53.09 pg/mL, 2.281 ± 52.90 pg/mL, 2.397 ± 53.72 pg/mL, respectively) significantly (Figure 3C).

NF-κB p65 levels in the INDO group measured significantly higher (224.7 ± 12.34 pg/mL) than in the control group (159 ± 13.31 pg/mL); *p* < 0.05). Only 250 mg/kg FA treatment decreased NF-κB levels significantly (156.5 ± 18.40 pg/mL; *p* < 0.05). These results are in line with the evaluation of TNF-α, IL-6 and MPO where the 250 mg/kg dose of FA showed the greatest reduction in the inflammation process (Figure 3D).

### 3.4. Histological Evaluation

**Figure 4:**
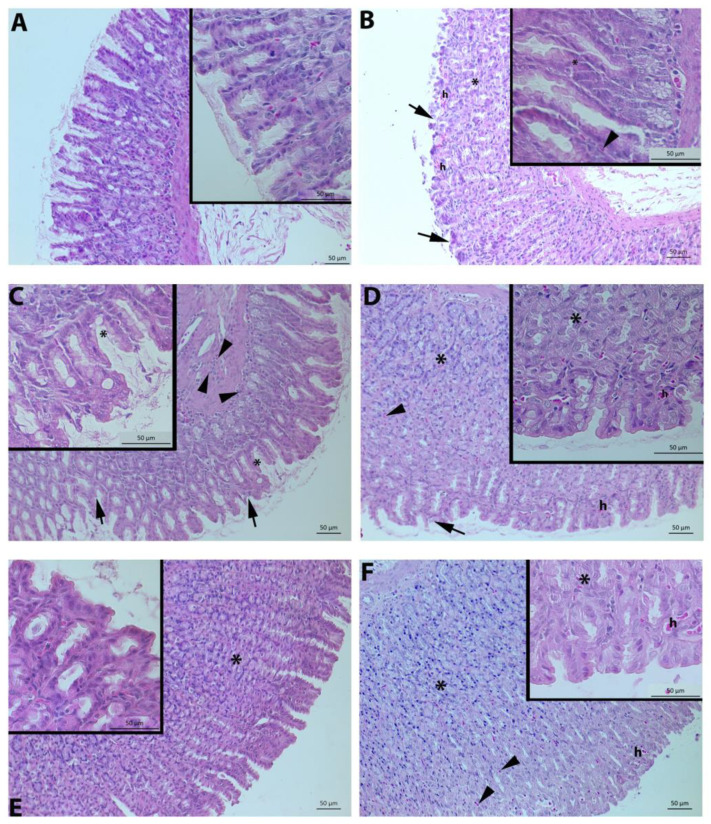
Representative light micrographs of gastric tissue samples. Normal morphology in gastric mucosa with surface epithelium and gland structure in the control group (**A**). Severe damage in the surface epithelium, glandular degeneration, hemorrhage, and inflammatory cell infiltration in the INDO group (**B**,**C**). Mild epithelial damage, glandular degeneration, and inflammatory cell infiltration in the FA100 group (**D**). Slight mucosal damage with mild glandular degeneration in the FA250 group (**E**). Moderate mucosal and glandular degeneration in the FA500 group (**F**). INDO: Indomethacin; FA100: Indomethacin+ Ferulic Acid 100 mg/kg; FA250: Indomethacin + Ferulic Acid 250 mg/kg; FA500: Indomethacin + Ferulic Acid 500 mg/kg. epithelial damage (arrow), inflammatory cell infiltration (arrowhead), hemorrhage (h) glandular degeneration (asterisk). H&E staining.

Under light microscopy, epithelial desquamation, mucosal hemorrhage, glandular damage, and inflammatory cell infiltration parameters were assessed. Normal morphology with surface epithelium and gastric glands was present in the control group (Figure 4A). On the other hand, indomethacin injection caused severe gastric tissue damage and apparent histopathological changes, including severe desquamation of surface epithelium, hemorrhage, glandular degeneration, and inflammatory cell infiltration (Figure 4B,C). Desquamation of surface epithelium, degeneration of the gastric gland, and inflammatory cell infiltration in the mucosa were obviously visible in the 100 mg/kg FA (Figure 4D). Mucosal degeneration, hemorrhage, and inflammatory cell infiltration were also detected in the FA500 group (Figure 4F). On the other hand, 250 mg/kg FA treatment attenuated damage induced by indomethacin and decreased markedly the infiltration of neutrophils and hemorrhage (Figure 4E). In addition, the FA250 group had a slight degeneration of the mucosa compared to the control group (Figure 1B).

**Figure 5:**
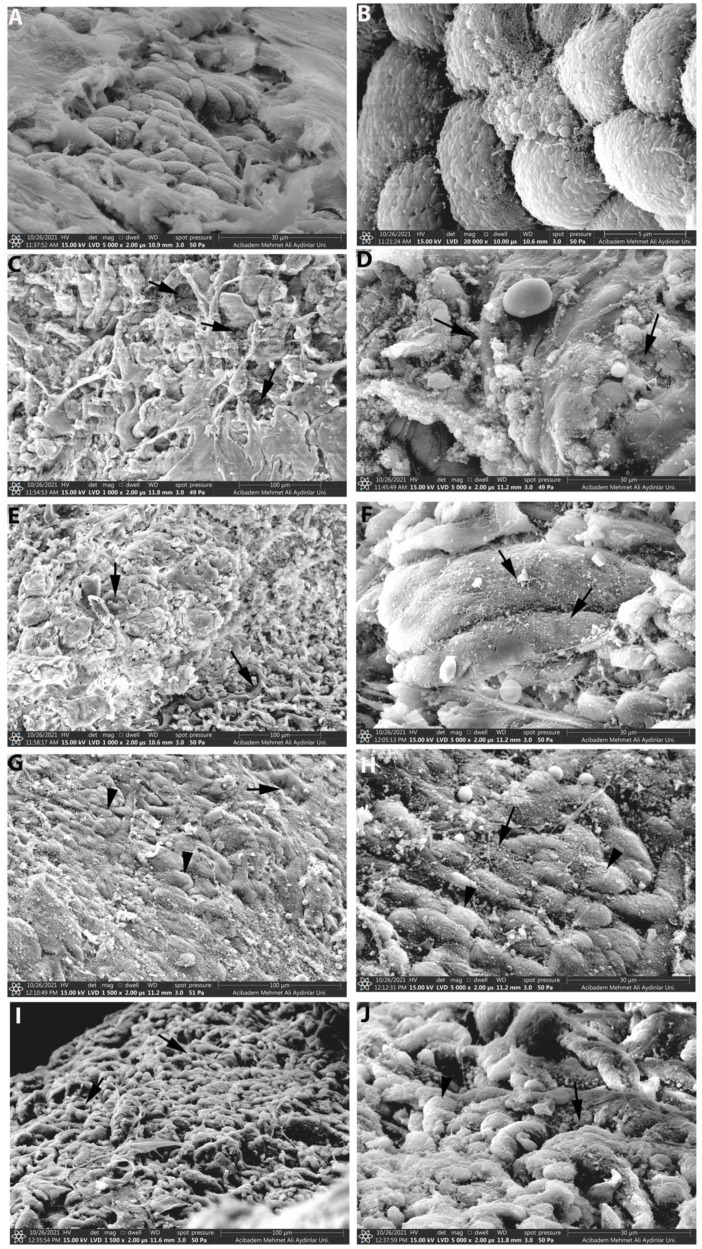
Representative electron micrographs of gastric tissue samples. Surface epithelium with normal topography in the control group (**A**,**B**). Damaged surface epithelium in the INDO group (**C**,**D**). Mild epithelial damage in the FA100 (**E**,**F**) group. Quite regular morphology in the FA250 group (**G**,**H**). Mild epithelial degeneration in the FA500 group (**I**,**J**). INDO: Indomethacin; FA100: Indomethacin+ Ferulic Acid 100 mg/kg; FA250: Indomethacin + Ferulic Acid 250 mg/kg; FA500: Indomethacin + Ferulic Acid 500 mg/kg epithelial damage (arrow), normal epithelial cells (arrowhead).

Stomach samples were also examined for surface epithelium integrity under the scanning electron microscope. The control group had steady surface epithelium (Figure 5A,B), while the surface epithelium was degenerated in the INDO group (Figure 5C,D). The damage to the surface epithelium caused by indomethacin improved in the FA250 group (Figure 5G,H), whereas the FA100 (Figure 5E,F) and FA500 (Figure 5I,J) groups were not different from the INDO group.

## 4. Discussion

In the present study, for the first time, we investigated the gastroprotective effects of FA (100 mg/kg, 250 mg/kg, and 500 mg/kg) on the indomethacin-induced gastric ulcer model in terms of biochemical and inflammatory parameters in rats. We showed that FA has antioxidant, anti-inflammatory, and anti-ulcer effects.

Due to the blocking of COX activity, indomethacin decreases the prostaglandin level. Additionally, it decreases mucus and HCO_3^−^_ secretion and mucosal blood flow. These effects also significantly contribute to changes in microvascular structures [21]. We used a semiquantitative scale for macroscopic scoring depending on the evaluation of the hemorrhages and petechial lesions in the gastric injury induced by indomethacin. In this context, ulceration and dark red-black bleeding areas were detected in the gastric mucosa of rats with gastric damage induced by indomethacin. All three doses of FA treatment improved gastric mucosal damage and lowered the macroscopic score. The 250 mg/kg dose of FA has shown a considerable decrease in damage score. This considerable increase in macroscopic score following indomethacin treatment might be attributed to an increase in ROS and inhibition of prostaglandin synthesis inhibition. FA attenuates macroscopic damage scores probably due to its antioxidant effects.

The MPO enzyme is localized in phagocytic cells and causes excessive production of ROS. It is a marker of regulation of neutrophil infiltration, which catalyzes the production of highly reactive HOCl from H_2_O_2_, which subsequently causes inflammation [22]. It was previously reported that indomethacin-induced mucosal damage induces an increase in mucosal MPO levels, suggesting that neutrophil infiltration plays a role in indomethacin-induced stomach injury [23]. Treatment with FA reduces tissue MPO activity in animals with nephrotoxicity [24]. In the current study, an increase in MPO activity was detected in the gastric tissues of rats with gastric damage induced by indomethacin. This finding indicates a considerable influx of neutrophils into the mucosa in response to the subcutaneous administration of indomethacin in rats, which is consistent with the earlier research. Compared to the INDO group, increased gastric MPO activity decreased with FA administration, which indicates that FA, as an anti-ulcer agent, acts by diminishing neutrophil adherence.

During the production of ATP through mitochondrial respiration, the oxygen molecule is reduced to water and ROS are produced [25]. The imbalance between free radical formation and scavenging capacity results in oxidative stress, which plays a role in the pathogenesis of gastric ulcers. These ROS cause damage to gastric tissue by damaging membranes and cellular macromolecules such as lipids, proteins, carbohydrates, and nucleic acids [26]. MDA is a lipid peroxidation product that is the most widely used indicator for determining oxidative stress in cell and tissue damage [27]. NSAIDs, including indomethacin, increase oxidative stress by enhancing lipid peroxidation and thus cause stomach damage [28]. Furthermore, it was shown that FA treatment reduced MDA levels in a streptozotocin-induced diabetes model [29]. In this study, tissue MDA levels were dramatically higher in gastric damage with ulcer induction compared with the control group. On the contrary, treatment with 250 mg/kg and 500 mg/kg doses of FA significantly diminished high MDA levels in ulcerated gastric tissue. FA’s capacity to scavenge ROS results in decreased levels of lipid oxidation, therefore helping to reduce oxidative gastrointestinal damage caused by ROS.

The body defends against the harmful effects of constantly produced ROS with the help of endogen antioxidants [30]. GSH is an endogenous antioxidant that helps preserve mucosal integrity and protect the gastric mucosa from free radical-induced tissue damage [31]. Based on previous research, indomethacin administration reduces GSH levels in stomach tissue [32]. In our study, GSH depleted in the gastric tissues of rats with gastric ulcers and FA treatment increased GSH levels, and the most effective dose was determined to be 250 mg/kg. Inhibition of GSH consumption in the stomach may constitute an important defense mechanism against oxidative stress-related gastric ulcers. Similar to our results, FA treatment prevents the depletion of tissue GSH levels in a hepatotoxicity model [33].

As one of the antioxidant enzymes that contributes to the enzymatic defense mechanisms, SOD protects the stomach tissue against damage by converting highly reactive O2-to less-reactive H_2_O_2_ as the first line of defense against ROS [31]. We know that a decrease in SOD expression occurs in gastric mucosal tissue after indomethacin administration [34]. FA treatment has been shown to increase tissue SOD levels in models of hepatotoxicity [35]. While indomethacin-induced stomach injury resulted in a drop in SOD levels, the antioxidant property of FA treatment improved SOD levels compared to the INDO group, as the most effective dose was 250 mg/kg. The reduction in SOD levels caused by indomethacin is consistent with prior research findings. Activating antioxidant mechanisms involving SOD in gastric tissues contributes to the preservation of structural and functional mucosal integrity against indomethacin [36]. At the same time, the FA significantly reduced the lipid peroxidation level in stomach tissue when compared to the INDO group, which indicates that the FA may exert its gastroprotective effect via an antioxidant mechanism.

Ferulic acid has the structural components of the 3-methoxy and 4-hydroxyl groups on the benzene ring, as well as the carboxylic acid group. These components either stabilize the resulting phenoxyl radical intermediate or even inhibit the free radical chain reaction [37]. In addition, ferulic acid increases the antioxidant enzyme activity, including SOD, glutathione peroxidase, and catalase. Thus, the cytoprotective effects of FA may be related to its structure and ability to improve antioxidant enzyme activity [38]. In the study, improvements in the anti-oxidant enzyme SOD and endogenous GSH levels were compatible with FA’s anti-oxidant properties.

Inflammation is a complex response to tissue damage that involves immune cells secreting proinflammatory cytokines, including TNF-α, IL-1β, and IL-6. The secretion of proinflammatory mediators and activation of the NF-κB signaling pathway play a critical role in the pathogenesis of gastric ulcers [39]. TNF-α is a proinflammatory cytokine released primarily by activated macrophages and it is considered a marker to assess gastric ulcer severity, closely related to the acute phase of inflammation and the degree of ulceration [40]. TNF-α and IL-1β have a synergistic effect and are associated with the acute phase of inflammation and the severity of a gastric ulcer [41]. In addition, IL-6 stimulates lymphocytes, macrophages, and neutrophils in the inflammatory region and triggers the oxidative pathway responsible for tissue damage during gastric ulcer. As suggested before, IL-6 levels increase in indomethacin-induced ulcers [42]. In this study, TNF-α, IL-1β, and IL-6 levels were higher in the INDO group compared to the control group. Compared to the INDO group, 250 mg/kg FA and 500 mg/kg FA doses significantly reduced TNF-α levels. Furthermore, only 250 mg/kg of FA treatment decreased tissue IL-6 levels compared to the INDO group, and FA had no significant effect on IL-1β levels. In addition, FA treatment has been shown to reduce serum TNF-α levels in a mouse model of high-fat diet-induced obesity [43] and serum IL-6 levels in formaldehyde-induced hepatotoxicity [44]. However, this is the first time that the inhibitory effects of FA treatment on gastric TNF-α and IL-6 levels have been demonstrated in an indomethacin-induced ulcer.

NF-κB, a crucial transcription factor, plays a vital role in the immune and inflammatory processes because it regulates the expression of various proinflammatory factors. There are four transcript variants of NF-κB encoding different isoforms, namely, p65, p105, p50, and p52 [45]. In resting conditions, NF-κB is retained in the cytosol as an inactive dimer bound to the NF-κB inhibitor (IκB) protein. When NF-κB is activated, the inhibitory complex IB is phosphorylated and degraded, which is controlled by the IB kinase (IKK), allowing the release of the NF-κB p65. The NF-κB p65 subunit is then translocated to the nucleus, where it stimulates the production of various inflammatory mediators [46]. Indomethacin activates NF-κB and induces the expression of several inflammatory genes, including TNF-α, IL-1β, and IL-6 [47]. The NF-κB signaling pathway is associated with the pathogenesis and progression of gastric ulcer formation, and its inhibition has protective functions in the development of gastric ulcers [48]. NF-κB p65 levels were measured in gastric tissue and NF-κB p65 was significantly higher in the INDO group than in the control group. Treatment with FA decreased NF-κB p65 expression compared to the INDO group. It was determined that 250 mg/kg FA revealed a substantial reduction in the gastric level of NF-κB p65 compared to the INDO group. Similar to our results, FA treatment has been shown to diminish NF-κB p65 expression in lipopolysacharide-induced acute kidney injury [49]. As a result, FA lowered the indomethacin-induced gastric ulcer damage by decreasing the proinflammatory cytokine levels via the NF-κB signal pathway. NF-κB may play a crucial role in protecting the stomach against gastric injury by regulating the expression of proinflammatory parameters [47]. Extracts obtained from *Lithraea molleoides* [50], *Baccharis dracunculifolia* [51], and *Pachira glabra leaves* [52], all of which contain ferulic acid, have been shown to have gastroprotective and anti-ulcer effects. It has been shown that *Pachira glabra* leaves also exert their effects on ethanol-induced gastric ulcer damage by reducing the levels of NF-κB and COX-2.

Indomethacin injection causes erosive and ulcerative gastric lesions with histopathological findings [53]. In our study, the control group exhibited normal morphology, but the indomethacin-induced group had a significantly higher score with severe damage in gastric gland morphology, local hemorrhage, and inflammatory cell infiltration in the mucosa. Three FA groups significantly decreased microscopic scoring compared to the INDO group. In addition, scanning electron microscopy data showed that the gastric tissues in the control group reflected the normal topography, while in the INDO group, there was damage to the surface epithelium and deterioration in the basal lamina structure in line with the previous findings [54]. The epithelial cells reflected equivalent to normal topography in scanning electron microscopy at a dose of 250 mg/kg FA, which provided a remarkable decrease in microscopic scoring against the damage caused by indomethacin.

## 5. Conclusions

This study is the first to report that FA protects the gastric mucosa against indomethacin-induced gastric mucosal damage. The protective effect of FA against gastric damage is related to its reducing effect on indomethacin-induced oxidative stress and inflammation. FA exhibits its gastroprotective effects via inhibiting neutrophil infiltration, suppressing lipid peroxidation, and modulating the antioxidant defense mechanisms. Moreover, it defends the stomach mucosa and plays a role in the structural integrity of the mucosa by inhibiting the NF-κB transcription factor, reducing damage at the tissue level. As a result, treatment with FA, especially the 250 mg/kg FA dose, has a marked gastroprotective effect against indomethacin-induced gastric ulcers. By means of its anti-ulcer properties derived from its antioxidant and anti-inflammatory activities, FA could be a promising therapeutic candidate for indomethacin-induced gastric ulcers.

## Data Availability

The data used to support the findings of this study are available from the corresponding author upon request.
